# On the performances of different IMRT treatment planning systems for selected paediatric cases

**DOI:** 10.1186/1748-717X-2-7

**Published:** 2007-02-15

**Authors:** Antonella Fogliata, Giorgia Nicolini, Markus Alber, Mats Åsell, Alessandro Clivio, Barbara Dobler, Malin Larsson, Frank Lohr, Friedlieb Lorenz, Jan Muzik, Martin Polednik, Eugenio Vanetti, Dirk Wolff, Rolf Wyttenbach, Luca Cozzi

**Affiliations:** 1 Oncology Institute of Southern Switzerland, Medical Physics Unit, Bellinzona, Switzerland; 2 Universitätsklinikum Mannheim, Klinik für Strahlentherapie und Radioonkologie, Mannheim, Germany; 3 Biomedical Physics, Radiooncology Dept, Uniklinik für Radioonkologie Tübingen, Tübingen, Germany; 4 Nucletron Scandinavia AB, Uppsala, Sweden; 5 RaySearch Laboratories, Stockholm, Sweden; 6 Ospedale Regionale Bellinzona e Valli, Radiology Dept, Bellinzona, Switzerland

## Abstract

**Background:**

To evaluate the performance of seven different TPS (Treatment Planning Systems: Corvus, Eclipse, Hyperion, KonRad, Oncentra Masterplan, Pinnacle and PrecisePLAN) when intensity modulated (IMRT) plans are designed for paediatric tumours.

**Methods:**

Datasets (CT images and volumes of interest) of four patients were used to design IMRT plans. The tumour types were: one extraosseous, intrathoracic Ewing Sarcoma; one mediastinal Rhabdomyosarcoma; one metastatic Rhabdomyosarcoma of the anus; one Wilm's tumour of the left kidney with multiple liver metastases. Prescribed doses ranged from 18 to 54.4 Gy. To minimise variability, the same beam geometry and clinical goals were imposed on all systems for every patient. Results were analysed in terms of dose distributions and dose volume histograms.

**Results:**

For all patients, IMRT plans lead to acceptable treatments in terms of conformal avoidance since most of the dose objectives for Organs At Risk (OARs) were met, and the Conformity Index (averaged over all TPS and patients) ranged from 1.14 to 1.58 on primary target volumes and from 1.07 to 1.37 on boost volumes. The healthy tissue involvement was measured in terms of several parameters, and the average mean dose ranged from 4.6 to 13.7 Gy. A global scoring method was developed to evaluate plans according to their degree of success in meeting dose objectives (lower scores are better than higher ones). For OARs the range of scores was between 0.75 ± 0.15 (Eclipse) to 0.92 ± 0.18 (Pinnacle^3 ^with physical optimisation). For target volumes, the score ranged from 0.05 ± 0.05 (Pinnacle^3 ^with physical optimisation) to 0.16 ± 0.07 (Corvus).

**Conclusion:**

A set of complex paediatric cases presented a variety of individual treatment planning challenges. Despite the large spread of results, inverse planning systems offer promising results for IMRT delivery, hence widening the treatment strategies for this very sensitive class of patients.

## Background

Radiation Therapy is administered to approximately one-half of the children affected by oncological pathologies to manage their disease [1]. The choice of available radiation treatments includes intensity-modulated radiotherapy (IMRT) that should therefore be investigated in the challenging field of paediatric radio-oncology.

IMRT has been proven, at least in planning studies, to improve conformal avoidance when compared to 3D conformal techniques (3DCRT) [2-7]. Improved dose distributions are generally expected to correlate with (significant) reduction of acute and late toxicity as already documented in paediatric radiation oncology by some authors, who reported low morbidity in children treated with IMRT [8-11]. As an example, in a cohort of 26 patients treated for medulloblastoma, the mean dose delivered to the auditory apparatus was 36.7 Gy for IMRT and 54.2 Gy for 3DCRT; 64% of the 3DCRT treated patients developed grade 3 to 4 hearing loss, compared to only 13% in the IMRT group [8].

Despite its potential, IMRT is not widely used in the paediatric field, and its introduction is significantly slower than for adults. Consequently, there is a substantial lack of knowledge on the late side effects of IMRT as pointed out in the review article of Rembielak [12]. The main limitation observed in this review is the publication of data of small series and short-term follow-up. In addition, the majority of studies investigated tumours located in the brain and CNS, with few other sites [8-10,13-15].

One of the major factors limiting the use of IMRT in paediatric oncology lies in the possible increase of radiation-induced secondary malignancies, caused mostly by the increased volume of patient receiving low dose levels. This effect derives from the generally increased number of fields entering from various angles and from a higher number of monitor units (MU) compared with 3DCRT, delivering higher leakage radiation estimated to be from 2 to 12 times higher than 3DCRT. However, this issue is controversial. Followill [16] showed that for 6 MV treatments the estimated likelihood of a fatal secondary cancer due to a 70 Gy treatment increased from 0.6% for wedged conventional treatment to 1.0% for IMRT, showing that 3DCRT is not significantly different from IMRT. Also Koshy [17] have found (in children treated for head-and-neck, brain, trunk, abdomen and pelvis) no significant differences in dose received by thyroid and breast glands when IMRT or 3DCRT were administered. Paediatric treatments are anyway delicate since enhanced radiation sensitivity is expected. Hall [18,19] showed that children are more sensitive than adults by a factor of 10; in addition, radiation scattered inside the patient is more significant in the small body of a child than in a larger adult body, and there is a genetic susceptibility of paediatric tissues to radiation-induced cancer. Therefore, there is a need of more clinical IMRT studies to assess the balance between the positive therapeutic effects and the risk of radiation-induced secondary malignancies.

The present study aimed to address the problem of IMRT in paediatric radiation oncology from a different point of view. Assuming that research activity in treatment planning or at clinical level shall be promoted, it is important to analyse if the tools available for IMRT are adequate and effective. A comparative study was conducted, similar to a previous investigation on breast cancer [20], on the most commonly available Treatment Planning Systems (TPS) to assess their respective performance and their potential limits in planning IMRT for some paediatric indications that were chosen as difficult to be treated optimally with 3DCRT. The rationale to develop and report a study like the present is multifactorial and is mainly based on the following pillars.

i) at present, very few studies, and probably none on paediatrics, exist addressing the issue of comparing different commercial planning systems for IMRT. The study on breast was the first published by this research group and aimed to prove (with a minimally acceptable set of five homogeneous patients) the adequacy of various TPS in terms of conformal avoidance, for a specific tumour side. Having proved that principle, it was felt necessary to expand the research on a different class of patients.

ii) with the new study we aimed to address the usability of the commercial TPS on pathologies which are more complicate in nature, rarer and more challenging such as pediatric cases where treatment planning requires particular skills and it is bounded by dose-limiting constraints often severely different from the ones applied to adults. As mentioned, literature is poor in this respect.

iii) in the field of paediatrics there is a generally weak knowledge about IMRT and, to complicate the problem, the variety of indications is huge and, at the limit, every individual patient presents peculiarities (given by the physiological variability in the evolutionary age) preventing easy generalisations. Therefore, rather than trying to identify one single pathology and a consistent cohort of patients, in the present study we preferred to identify a (small) group of complicate cases, one case per indication, but all of them presenting specific planning challenges. On the other side, it was decided to limit the number of cases to present in order to minimise data presentation considering the results qualitatively sufficient to prove the aims.

iv) the study aimed at understanding if systems were keeping the reliability shown for breast also under conditions uncommon and distant from those generally used in IMRT planning and likely not tested in the development and qualification phases.

The strategy described above, allowed testing IMRT capabilities of routinely available commercial TPS under a range of rather extreme (although rare) conditions. In this respect, the specific choice of indications, and the actual status of the selected case, does not limit or affect the potential of investigating complicate situations that could be used as templates for similar cases. Clinical questions (like outcome and toxicity) should be addressed in properly designed clinical trials and are not subjects of comparative planning studies.

## Methods

Four paediatric patients, affected by different types of cancer, were chosen. The tumour types were: one extra osseous, intrathoracic Ewing Sarcoma; one mediastinal Rhabdomyosarcoma; one Rhabdomyosarcoma of the anus with intrapelvic, inguinal and osseous metastases; one Wilm's tumour of the left kidney with multiple liver metastases. In table 1 a summary of the diagnosis, dose prescriptions, and planning objectives (PObj) for organs at risk (OAR) is presented. For all cases except patient 4, the treatment was structured in two courses, with two different planning target volumes (PTV): PTV1 being the elective and PTV2 the boost volumes. The PObj concerning OARs refer mainly to the report of the National Cancer Institute [21,22]. To avoid scaling effects due to optimisation [20], dose was normalised to the mean PTV value.

**Table 1:** Main characteristics of patients and treatment.

	**Patient 1**	**Patient 2**	**Patient 3**	**Patient 4**
**Patient**	Male, 12 y.o.	Female, 8 y.o.	Female, 13 y.o.	Female, 8 y.o.

**Diagnosis**	Ewing Sarcoma extraosseous, intrathoracic	Rhabdomyosarcoma mediastinum, stage III	Rhabdomyosarcoma anus. Metastasis lymphnodes intrapelvic, inguinal and osseous	Wilm's tumour of the left kidney. (Multiple lung metastasis). Multiple liver metastasis
**Status**	After chemotherapy + surgery + chemotherapy	After chemotherapy	After chemotherapy	After chemotherapy + left nefrectomy + chemo-radiotherapy for lung metastasis
**Radiotherapy dose prescription**	Total = 54.4 Gy,	Total = 50.4 Gy,	Total = 50.4 Gy,	Total = 18 Gy,
	1.6 Gy/fraction	1.8 Gy/fraction	1.8 Gy/fraction	1.2 Gy/fraction
	2 fractions/day	1 fraction/day	1 fraction/day	1 fraction/day
	I course (PTV1) = 44.8 Gy	I course (PTV1) = 45 Gy	I course (PTV1) = 45 Gy	
	II course(PTV2) = 9.6 Gy (boost, excludes surgical scar)	II course (PTV2) = 5.4 Gy (boost)	II course (PTV2) = 5.4 Gy (boost, excludes the two inguinal nodes regions)	
**Target volumes**	PTV1 = 564 cm^3^	PTV1 = 109 cm^3^	PTV1 = 618 cm^3^	PTV1 = 1234 cm^3^
	PTV2 = 549 cm^3^	PTV2 = 72 cm^3^	PTV2 = 193 cm^3^	
**Organs at risk dose objectives**	Lung^1 ^< 15 Gy	Lung^1 ^< 15 Gy	Rectum^1 ^< 40 Gy	Kidney^1 ^< 10 Gy
	Heart^1 ^< 30 Gy	Heart^1 ^< 30 Gy	Bladder^1 ^< 30 Gy	
	Vertebra^1 ^< 20 Gy	Vertebra^1 ^< 20 Gy	Uterus^1 ^< 20 Gy	
	Spinal cord^2 ^< 45 Gy	Spinal cord^2 ^< 45 Gy	Femural heads^1 ^< 20 Gy	
**Beam arrangement**	6 fields. (both courses) Gantry angles: 180, 165, 125, 90, 60, 340	7 fields. (both courses) Gantry angles: 0, 30, 100, 130, 230, 260, 330	I course: 7 fields. Gantry angles: 0, 51, 103, 154, 206, 257, 308	5 fields Gantry angles: 0, 72, 144, 216, 288
			II course: 5 fields: Gantry angles: 0, 72, 125, 235, 288	

1: mean dose; 2: maximum dose

Datasets were distributed among participants in DICOM (CT images) and DICOM-RT (contours of volumes of interest – VOIs) format as defined at the reference centre (Bellinzona, Switzerland).

Seven TPS with inverse planning capabilities were compared. Information on release used and main references for dose calculation and optimisation algorithms are reported in table 2. All TPS, except Hyperion, are commercial systems. Pinnacle^3 ^implemented two optimisation methods: one related to physical quantities and the other to a combination of physical and 'biological' (Equivalent Uniform Dose, EUD) quantities and was therefore considered twice. Hyperion combined 'biological' optimisation with a Monte Carlo (MC) engine. All the other TPS have optimisation engines which rely on physical optimisation only and dose calculation was performed using either pencil beam (PB) or convolution/superposition algorithms such as the Collapsed Cone (CC) or the Anisotropic Analytical Algorithm (AAA) or MonteCarlo (MC). All TPS (except Eclipse and KonRad) supported only static segmental (step-and-shoot) IMRT; Eclipse plans in the present study used dynamic (sliding window) MLC sequencing. The number of intensity levels (IL) used by the static systems to discretise individual beam fluence was generally 10. For Corvus IL was set to 3, but it is an aperture based system with manual segment generation and inverse optimisation of the segment weights. For Hyperion, the segmentation process does not use ILs, rather a set of constraints such as segment size, dose per segment and total number of segments. For OMP and Pinnacle^3 ^the total number of segments, the segment size and the minimum MU per segment are the set parameters.

**Table 2:** TPS characteristics and references

**TPS, release**		**Calculation alg.**	**Optimisation alg.**	**References**
Corvus, 5.0	Corvus	Pencil beam	Simulated annealing	[25]
Eclipse, 7.5.14.3	Eclipse	Anisotropic Analytical Algorithm (AAA)	Conjugated gradient	[26,27,28,29,30,31,32]
Hyperion, 2.1.4	Hyperion	Monte Carlo	Conjugate gradient	[33,34,35,36]
KonRad, 2.2.18	KonRad	Pencil beam	Conjugate gradient	[37,38]
Oncentra Master Plan, 1.5	OMP	Pencil beam	Conjugate gradient	[39,40]
Pinnacle^3 ^EUD, 7.4f	PinnEUD	Collapsed cone	Gradient based, sequential quadratic programming	[41,42,43,44]
Pinnacle^3 ^Phys, 7.4f	PinnPhy	Collapsed cone	Gradient based, sequential quadratic programming	[42,45,46]
PrecisePLAN, 2.03	Precise	Pencil beam	Cimmino	[47]

A set of procedural guidelines was defined including specifications of the PObj's to fulfil. Given the specifics of each TPS, the choice of numerical objectives translating the PObj into e.g. dose-volume constraints was not fixed. Also 'dummy' volumes, steering the optimisation engines to improve results, were allowed to compare the 'best' plans under given conditions [20]. To avoid variability in the results due to different beam arrangements, the number of fields and the beam geometry were fixed. Bolus was allowed if required. All plans were designed for 6 MV photon beams using multileaf collimators with 80 or 120 leaves. The three following objectives should be achieved: *i) *target coverage (min. dose 90%, max. dose 107%), *ii) *OAR sparing to at least the limits stated in table 1, *iii) *sparing of healthy tissue (HTis, defined as the CT dataset patient volume minus the volume of the largest target). The dose limits on OARs and HTis were strengthened by the additional requirement to minimise the volumes involved. No specific model for the calculation of the risk of secondary cancer induction was applied because of no consensus about their value. Hence, the analysis was limited to the evaluation of physical quantities. Every TPS was required, using whichever method, to minimise the involvement of HTis. The dose constraints reported in table 1 are specific to paediatric cases and more restrictive than the corresponding for adults and all were derived from specific literature publications.

The cases and indications were selected in order to obtain a minimal set of complicate planning situations with specific challenges to resolve to test TPS capabilities.

For patient 1 the main challenges were: the target was adjacent to the spinal cord, partially inside the lung with a long scar (about 5 cm) generating a secondary target volume, separated from the main one, smaller in volume and located along the thoracic wall but requiring simultaneous irradiation. Complementary to these geometrical conditions, there is a generic need, in paediatrics, to generate rather symmetric irradiation of the body (in this case the vertebrae) to prevent potential risks of asymmetric growth.

For patient 2, the location of the target in the mediastinum would be relevant in terms of large dose baths in the lung (and eventually breast) regions.

For patient 3, the target volume was divided into three unconnected regions (the anal volume and the two inguinal node regions) with organs at risk generally positioned in-between the three targets (as uterus, bladder and rectum).

For patient 4, the target volume was given by the entire liver and the main organ at risk was the right kidney with a low tolerance, located proximal/adjacent to the target. The sparing of this kidney had a very high priority since the patient underwent left nephrectomy.

For patients 1, 2 and 3, treatment plans were generated for two separate treatment courses and for the complete treatment, as the sum of partial plans according to dose prescriptions reported in table 1. In no case was the concept of simultaneous integrated boost (SIB) applied. All TPS, except KonRad (in the implementation used although in principle possible), were able to produce the summed plan; for KonRad, only the mean doses to the VOIs were used in the analysis of the entire treatment since the sum of the mean doses in a VOI is equal to the mean dose of the summed plan in that VOI. The maximum point dose reported for the entire treatment for KonRad plans was recorded as the sum of the two separate plan maximum doses, even if this value could be overestimated (does not take into account the actual location of the individual plan maxima).

The TPS can be divided into two families: a first, where the two courses are planned independently (Corvus, Eclipse, KonRad) and a second, where the plans for the second course are optimised based upon knowledge of the dose distribution already "accumulated" in the first course (Hyperion, OMP, Pinnacle^3^, Precise). In principle, KonRad could belong to the second family, but in the present study it was not the case.

The number of MU/Gy has been investigated since in pediatric radiation oncology this is a highly relevant issue in terms of possible induction of secondary malignancies. MU values from the different TPS were normalised to a virtual output of 1 Gy for 100 MU, 10 × 10 cm^2 ^field, SSD = 90 cm and 10 cm depth (isocentre).

### Evaluation tools

The analysis was based on isodose distributions and on physical DVHs of PTVs, OARs and HTis. From DVHs, the following parameters were compared: D_x _(the dose received by x% of the volume); V_y _(the volume receiving at least y dose (in percentage of the prescribed dose or in Gy)); mean dose; maximum and minimum point doses; maximum and minimum significant doses defined as D_1% _and D_99% _respectively, and standard deviation (SD).

For HTis we also report the volume receiving at least 10 Gy normalised to the elective PTV (nV_10 Gy_) to assess the relative extent of irradiation at low dose levels.

A Conformity Index (CI) was defined for each PTV and treatment course as the ratio of the volume receiving 90% of the dose prescribed for this specific volume and the PTV itself.

Finally, to introduce a plan ranking, a 'goodness' parameter was defined for OARs (including HTis) and PTVs:

Score(OAR)=1nOAR∑n(Valplan/PObj)OARn     (1)
 MathType@MTEF@5@5@+=feaafiart1ev1aaatCvAUfKttLearuWrP9MDH5MBPbIqV92AaeXatLxBI9gBaebbnrfifHhDYfgasaacH8akY=wiFfYdH8Gipec8Eeeu0xXdbba9frFj0=OqFfea0dXdd9vqai=hGuQ8kuc9pgc9s8qqaq=dirpe0xb9q8qiLsFr0=vr0=vr0dc8meaabaqaciaacaGaaeqabaqabeGadaaakeaacqWGtbWucqWGJbWycqWGVbWBcqWGYbGCcqWGLbqzcqGGOaakcqWGpbWtcqWGbbqqcqWGsbGucqGGPaqkcqGH9aqpdaWcaaqaaiabigdaXaqaaiabd6gaUjabd+eapjabdgeabjabdkfasbaadaaeqbqaamaabmaabaWaaSGbaeaacqWGwbGvcqWGHbqycqWGSbaBdaWgaaWcbaGaemiCaaNaemiBaWMaemyyaeMaemOBa4gabeaaaOqaaiabdcfaqjabd+eapjabdkgaIjabdQgaQbaaaiaawIcacaGLPaaadaWgaaWcbaGaem4ta8KaemyqaeKaemOuai1aaSbaaWqaaiabd6gaUbqabaaaleqaaaqaaiabd6gaUbqab0GaeyyeIuoakiaaxMaacaWLjaWaaeWaaeaacqaIXaqmaiaawIcacaGLPaaaaaa@5BC2@

Score(PTV)=1nPTV∑n(Valplan/PObj)PTVn     (2)
 MathType@MTEF@5@5@+=feaafiart1ev1aaatCvAUfKttLearuWrP9MDH5MBPbIqV92AaeXatLxBI9gBaebbnrfifHhDYfgasaacH8akY=wiFfYdH8Gipec8Eeeu0xXdbba9frFj0=OqFfea0dXdd9vqai=hGuQ8kuc9pgc9s8qqaq=dirpe0xb9q8qiLsFr0=vr0=vr0dc8meaabaqaciaacaGaaeqabaqabeGadaaakeaacqWGtbWucqWGJbWycqWGVbWBcqWGYbGCcqWGLbqzcqGGOaakcqWGqbaucqWGubavcqWGwbGvcqGGPaqkcqGH9aqpdaWcaaqaaiabigdaXaqaaiabd6gaUjabdcfaqjabdsfaujabdAfawbaadaaeqbqaamaabmaabaWaaSGbaeaacqWGwbGvcqWGHbqycqWGSbaBdaWgaaWcbaGaemiCaaNaemiBaWMaemyyaeMaemOBa4gabeaaaOqaaiabdcfaqjabd+eapjabdkgaIjabdQgaQbaaaiaawIcacaGLPaaadaWgaaWcbaGaemiuaaLaemivaqLaemOvay1aaSbaaWqaaiabd6gaUbqabaaaleqaaaqaaiabd6gaUbqab0GaeyyeIuoakiaaxMaacaWLjaWaaeWaaeaacqaIYaGmaiaawIcacaGLPaaaaaa@5C54@

where the sum is extended to the number of evaluated OARs or PTVs (nOAR or nPTV), Val_plan _is, for each chosen parameter (one for each VOI, e.g. mean dose to the lung), the value found after DVH analysis of the sum plans; PObj are the relative plan objectives as in table 1. For HTis the V_10 Gy _parameter was chosen and, as PObj, the mean value of the parameter over all the TPS for each patient was used. The sum is normalised to the number of OARs or PTVs used. For PTVs, the Score analyses the fraction of volume receiving less than the 90% or more than the 107% of the prescribed dose in the first course plan and, for the boost, it analyses the data of the summed plans. In this way, the TPS of the second family are not penalised. According to the definition, the Score should be as low as possible and smaller than 1.

In the evaluation phase, plans were considered as acceptable if respecting (or minimally violating) the planning objectives and plans with lower scores were considered preferable.

## Results

**Figure 1:**
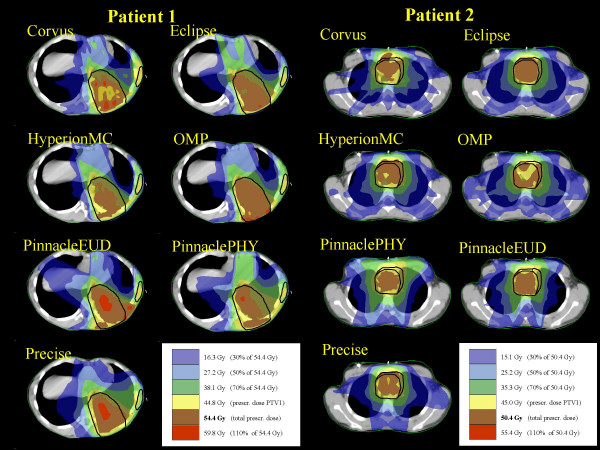
Dose distributions of the summed plan (overall treatment) for Patient 1 and Patient 2.

**Figure 2:**
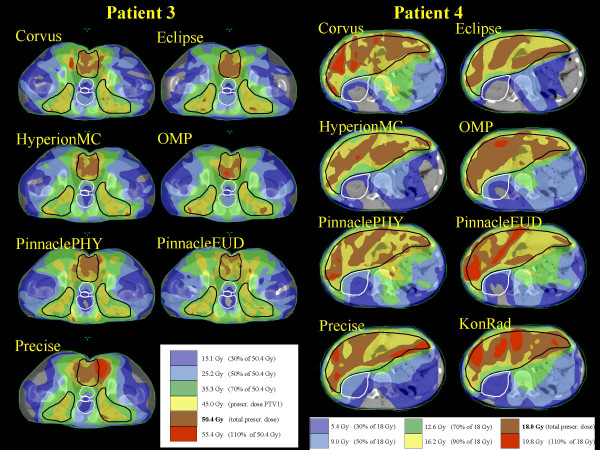
Dose distributions of the summed plan (overall treatment) for Patient 3 and Patient 4.

Figures 1 and 2 present, for a representative CT image, the dose distribution for the four patients, the PTVs shown in black and some relevant OARs in white. Data are reported for the total plan (i.e. sum of plans for PTV1 and PTV2 for the first 3 patients).

**Figure 3:**
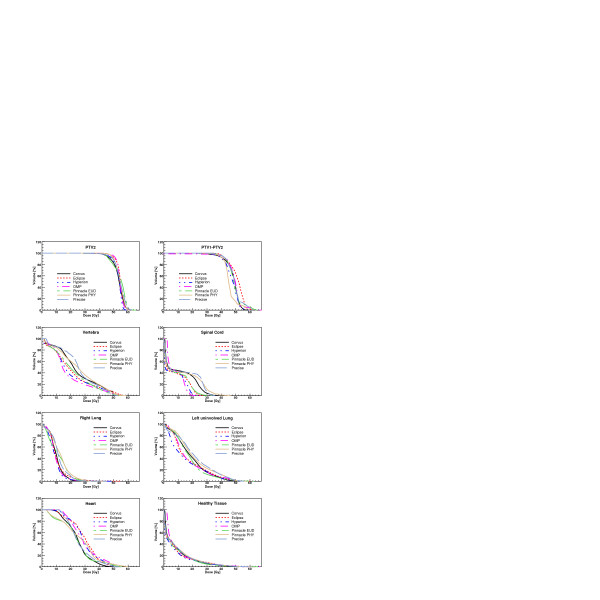
Dose-Volume Histograms for targets and all organs at risk for Patient 1. Data refer to the complete treatment.

**Figure 4:**
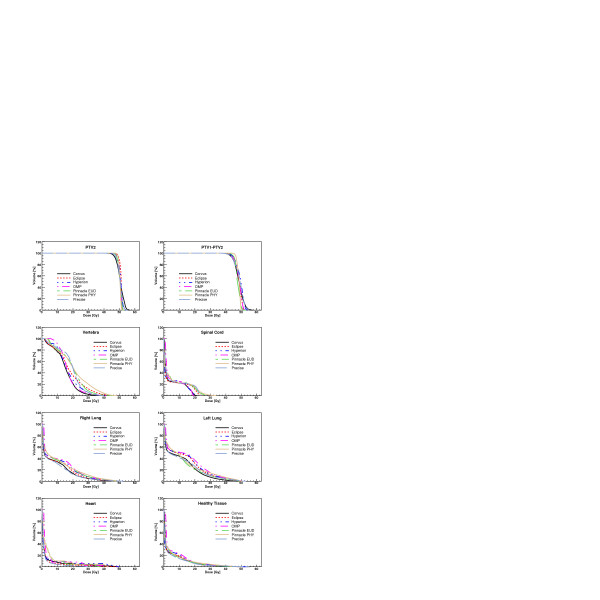
Dose-Volume Histograms for targets and all organs at risk for Patient 2. Data refer to the complete treatment.

**Figure 5:**
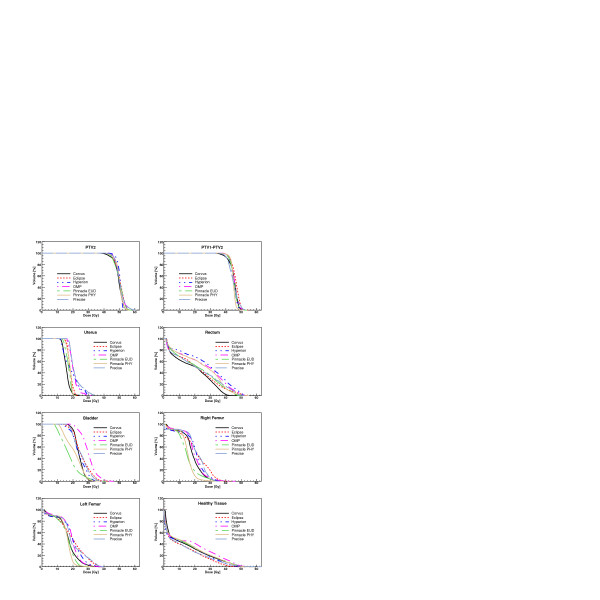
Dose-Volume Histograms for targets and all organs at risk for Patient 3. Data refer to the complete treatment.

**Figure 6:**
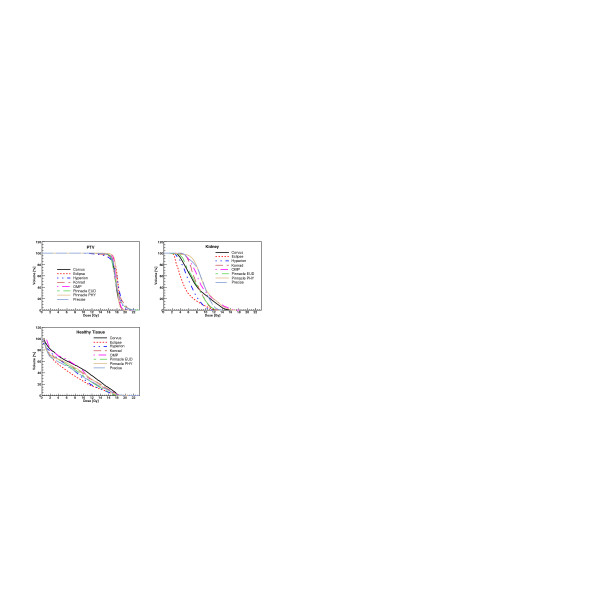
Dose-Volume Histograms for the target and all organs at risk for Patient 4.

Figures 3, 4, 5, 6 show the DVH of PTV2 (PTV for patient 4) and for the involved OAR for the total treatment for each patient and for all TPS.

From the dose distribution figures it is possible to qualitatively appraise the different degrees of conformal avoidance, the extension of the low dose areas, the degree of uniformity of doses within the PTVs and the potential presence of hot spots.

Table 3 presents for all OARs, PTVs and HTis, for all patients and for the most relevant parameters, the PObj and the average values computed over all the TPS. Uncertainty is given at one standard deviation (SD). Data for OARs are given for the total plans while for the first three patients PTV data are given for the two courses separately and, for PTV2 only, also for the total treatment (PTV2 (total)).

**Table 3:** Global results from total dose plan analysis. For each patient and parameter the average over all the planning systems is shown, uncertainties are reported at 1 standard deviation.

		Objectives	Patient 1	Patient 2	Patient 3	Patient 4
*PTV1*	Prescription	-	44.8	45.0	45.0	18.0
	D_1% _Gy	Prescript	50.4 ± 0.8	48.1 ± 1.5	50.2 ± 1.2	20.5 ± 0.9
	D_99% _Gy	Prescript	35.3 ± 2.2	40.5 ± 1.8	38.7 ± 1.3	14.4 ± 1.5
	V_90% _%	100%	93.6 ± 2.6	98.4 ± 1.6	96.3 ± 2.5	96.7 ± 2.3
	V_107% _%	0%	10.8 ± 5.4	2.7 ± 4.4	8.6 ± 4.9	9.4 ± 6.3
	SD Gy	0 Gy	3.0 ± 0.5	1.5 ± 0.7	2.4 ± 0.5	1.1 ± 0.3
	CI	1.0	1.14 ± 0.08	1.28 ± 0.10	1.58 ± 0.26	1.26 ± 0.14
*PTV2*	Prescription	-	9.6	5.4	5.4	-
	D_1% _Gy	Prescript	11.4 ± 1.5	6.1 ± 0.6	6.6 ± 1.4	-
	D_99% _Gy	Prescript	6.1 ± 2.5	4.3 ± 0.7	3.9 ± 1.6	-
	V_90% _%	100%	88.4 ± 10.9	91.8 ± 10.8	89.6 ± 16.9	-
	V_107% _%	0%	18.6 ± 18.5	14.2 ± 16.5	14.6 ± 19.7	-
	SD Gy	0 Gy	1.1 ± 1.0	0.4 ± 0.3	0.6 ± 0.7	-
	CI	1.0	1.07 ± 0.15	1.37 ± 0.16	1.33 ± 0.13	-
*PTV2 (total)*	Prescription	-	54.4	50.4	50.4	-
	Mean Gy	Prescript.	54.1 ± 0.5	50.6 ± 0.2	50.2 ± 0.1	-
	Max pt Gy	Prescript	63.5 ± 2.7	54.2 ± 2.0	57.3 ± 2.1	-
	D_1% _Gy	Prescript	60.3 ± 1.3	53.1 ± 1.3	55.7 ± 1.6	-
	D_99% _Gy	Prescript	42.7 ± 1.4	46.8 ± 1.4	43.1 ± 1.3	-
	V_90% _%	100%	91.3 ± 4.5	99.6 ± 0.6	95.4 ± 2.3	-
	V_107% _%	0%	10.3 ± 7.6	1.4 ± 3.2	7.1 ± 3.9	-
*Vertebra*	Mean Gy	<20 Gy	22.8 ± 2.5	18.0 ± 1.8	-	-
*Spinal Cord*	Max pt Gy	<45 Gy	33.3 ± 7.2	25.1 ± 4.7	-	-
	D_1% _Gy	Minimise	29.0 ± 7.8	23.1 ± 3.9	-	-
*Right Lung*	Mean Gy	< 15 Gy	10.8 ± 1.3	9.6 ± 1.5	-	-
	V_20 Gy _%	Minimise	7.6 ± 4.4	18.8 ± 4.1	-	-
*Left (uninv.) Lung*	Mean Gy	< 15 Gy	18.5 ± 2.2	12.6 ± 1.5	-	-
	V_20 Gy _%	Minimise	38.3 ± 8.1	27.9 ± 6.0	-	-
*Heart*	Mean Gy	<30 Gy	27.4 ± 2.6	3.9 ± 0.9	-	-
	Max pt Gy	Minimise	55.1 ± 2.4	43.2 ± 6.9	-	-
	D_1% _Gy	Minimise	51.4 ± 3.6	39.8 ± 7.8	-	-
*Uterus*	Mean Gy	<20 Gy	-	-	18.9 ± 1.8	-
	V_20 Gy _%	Minimise	-	-	26.2 ± 20.6	-
*Rectum*	Mean Gy	<40 Gy	-	-	23.6 ± 3.1	-
	V_40 Gy _%	Minimise	-	-	15.3 ± 7.7	-
*Bladder*	Mean Gy	<30 Gy	-	-	24.4 ± 3.6	-
	V_30 Gy _%	Minimise	-	-	16.9 ± 15.8	-
*Right Femur*	Mean Gy	<20 Gy	-	-	19.2 ± 3.2	-
	Max pt Gy	Minimise	-	-	40.7 ± 3.6	-
*Left Femur*	Mean Gy	<20 Gy	-	-	18.7 ± 2.6	-
	Max pt Gy	Minimise	-	-	38.4 ± 4.0	-
*Kidney*	Mean Gy	<10 Gy	-	-	-	7.9 ± 1.6
	Max pt Gy	Minimise	-	-	-	15.0 ± 2.3
	D_1% _Gy	Minimise	-	-	-	13.7 ± 2.0
	V_5 Gy _%	Minimise	-	-	-	83.6 ± 20.3
*Healthy tissue*	Mean Gy	Minimise	8.2 ± 0.7	4.6 ± 0.7	13.7 ± 1.7	7.5 ± 0.8
	Max pt Gy	Minimise	61.6 ± 5.3	52.2 ± 2.2	57.0 ± 3.8	20.8 ± 1.7
	V_10 Gy _cm^3^	Minimise	2700 ± 300	1060 ± 170	3620 ± 240	1450 ± 300
	nV_10 Gy_	Minimise	4.8 ± 0.6	9.5 ± 1.7	5.8 ± 0.4	1.2 ± 0.3
	V_90% _cm^3^	Minimise	74 ± 30	24 ± 8	189 ± 120	334 ± 122

Table 4 reports the averages, computed over the four patients and over all the PTVs (analysing the single plans), of the parameters expressing the degree of target coverage for all the TPS. For D_1% _and D_99% _the data are reported as percentage of the prescribed dose for each PTV.

**Table 4:** Global results on PTVs from dose plan analysis. For each TPS the averages over all the patients and target volumes are shown, uncertainties are reported at 1 standard deviation. D_1% _and D_99% _are reported as percentage of the prescribed dose.

	Objective	Corvus^1^	Eclipse^1^	Hyperion^2^	KonRad^1^	OMP^2^	PinnEUD^2^	PinnPhy^2^	Precise^2^
D_1% _%	100	112 ± 2	107 ± 4	109 ± 4	113 ± 6	111 ± 5	126 ± 24	116 ± 13	114 ± 7
D_99% _%	100	81 ± 6	87 ± 5	82 ± 9	78 ± 10	87 ± 4	68 ± 31	77 ± 22	83 ± 7
V_90% _%	100%	93.8 ± 3.7	97.1 ± 2.6	95.2 ± 2.9	96.6 ± 2.0	97.8 ± 1.2	83.8 ± 18.5	88.4 ± 12.6	95.6 ± 3.3
V_107% _%	0%	13.3 ± 6.5	3.9 ± 5.6	5.9 ± 4.8	9.6 ± 5.7	4.6 ± 3.5	24.1 ± 21.7	18.7 ± 20.9	10.2 ± 6.9
SD Gy	0 Gy	1.63 ± 1.39	1.19 ± 1.15	1.37 ± 1.08	1.44 ± 1.12	1.14 ± 1.02	1.87 ± 0.88	1.26 ± 0.66	1.54 ± 1.36
CI	1.00	1.29 ± 0.29	1.16 ± 0.09	1.34 ± 0.24	1.26 ± 0.14	1.35 ± 0.16	1.28 ± 0.30	1.32 ± 0.30	1.31 ± 0.14

^1^: TPS belonging to the first family type

^2^: TPS belonging to the second family type

Tables 5, 6, 7, 8 present for each patient the same parameters with the findings for each TPS.

**Table 5:** Results from dose plan analysis (total treatment) for Patient 1

	Obj	Corvus^1^	Eclipse^1^	Hyperion^2^	KonRad^1^	OMP^2^	PinnEUD^2^	PinnPhy^2^	Precise^2^
*PTV1 (44.8 Gy)*									
D_1% _Gy	Prescript	51.4	50.4	49.3	50.7	50.6	50.4	49.1	51.3
D_99% _Gy	Prescript	34.2	36.7	32.9	33.2	36.8	37.9	37.8	32.7
V_90% _%	100%	89.8	93.7	91.6	92.7	96.7	96.4	96.3	91.6
V_107% _%	0%	18.9	10.5	7.7	14.0	6.4	7.3	4.1	17.1
SD Gy	0 Gy	3.7	2.7	3.2	3.3	2.7	2.4	2.3	3.5
CI	1.0	1.03	1.10	1.06	1.13	1.24	1.20	1.24	1.10
									
*PTV2 (9.6 Gy)*									
D_1% _Gy	Prescript	11.1	10.3	10.6	10.5	10.8	14.4	13.0	10.7
D_99% _Gy	Prescript	6.6	8.3	7.5	5.8	7.8	0.8	4.5	7.3
V_90% _%	100%	87.9	97.7	94.4	96.8	96.1	69.3	74.1	91.2
V_107% _%	0%	22.6	1.2	6.2	4.6	6.9	53.6	37.5	16.5
SD Gy	0 Gy	0.9	0.4	0.6	0.6	0.6	3.3	1.7	0.7
CI	1.0	0.94	1.17	1.18	1.13	1.24	0.82	0.91	1.14

*PTV2 (54.4 Gy)*									
Mean Gy	54.4 Gy	53.7	54.5	53.1	54.4	53.9	54.3	54.3	54.3
Max pt Gy	Prescript	64.3	63.7	59.8	62.6	68.3	65.8	60.9	62.6
D_1% _Gy	Prescript	60.8	60.2	57.6	-	60.2	61.5	60.1	61.6
D_99% _Gy	Prescript	41.0	45.3	40.2	-	44.7	41.9	45.7	40.0
V_90% _%	100%	88.2	94.8	89.8	-	96.5	83.3	94.2	91.9
V_107% _%	0%	10.7	8.1	0.4	-	3.5	23.1	9.7	16.3

*Vertebra*									
Mean Gy	< 20 Gy	24.1	21.2	20.1	24.1	19.4	21.5	25.6	26.0
									
*Spinal Cord*									
Max pt Gy	< 45 Gy	38.0	27.8	23.4	35.1	27.4	33.2	46.2	35.6
D_1% _Gy	Minimise	34.6	24.6	21.6	-	19.5	28.2	41.3	33.4
									
*Right Lung*									
Mean Gy	< 15 Gy	10.1	10.4	9.0	11.1	9.8	11.6	13.0	11.6
V_20 Gy _%	Minimise	3.6	7.4	3.1	-	3.7	11.4	14.2	9.9
									
**Left uninv. Lung**									
Mean Gy	< 15 Gy	18.8	17.1	15.1	17.9	17.7	17.8	21.4	21.8
V_20 Gy _%	Minimise	42.0	32.2	30.0	-	31.3	36.0	45.7	50.9
									
*Heart*									
Mean Gy	< 30 Gy	24.8	29.1	28.8	31.5	29.3	23.8	26.0	26.0
Max pt Gy	Minimise	52.2	53.7	53.4	56.5	55.5	55.7	60.0	53.6
D_1% _Gy	Minimise	45.7	50.0	50.4	-	53.1	52.9	57.4	50.0
									
**Healthy tissue**									
Mean Gy	Minimise	8.3	7.9	7.0	8.6	9.2	7.6	8.5	8.4
Max pt Gy	Minimise	51.8	63.6	59.6	65.4	68.9	65.1	59.7	58.8
V_10 Gy _cm^3^	Minimise	2810	2340	2310	-	2640	2770	3020	3040
nV_10 Gy_	Minimise	4.9	4.1	4.0	-	4.9	4.7	5.2	5.6
V_90% _cm^3^	Minimise	31	86	52	-	127	71	77	78

^1^: TPS belonging to the first family type

^2^: TPS belonging to the second family type

**Table 6:** Results from dose plan analysis (total treatment) for Patient 2

	Obj	Corvus^1^	Eclipse^1^	Hyperion^2^	KonRad^1^	OMP^2^	PinnEUD^2^	PinnPhy^2^	Precise^2^
*PTV1 (45 Gy)*									
D_1% _Gy	Prescript	50.2	46.5	48.3	50.2	47.2	47.1	46.5	48.4
D_99% _Gy	Prescript	38.6	40.7	38.9	38.9	41.2	42.2	43.7	39.9
V_90% _%	100%	96.0	99.2	96.3	98.0	99.5	99.9	100.0	98.4
V_107% _%	0%	12.5	0.0	1.4	5.7	0.0	0.1	0.1	1.9
SD Gy	0 Gy	2.5	1.3	1.9	2.1	1.2	0.9	0.6	1.8
CI	1.0	1.18	1.17	1.30	1.21	1.41	1.20	1.43	1.31
									
*PTV2 (5.4 Gy)*									
D_1% _Gy	Prescript	6.2	5.5	5.9	5.8	5.8	7.3	6.6	6.0
D_99% _Gy	Prescript	4.5	5.0	4.6	4.2	4.7	3.0	3.6	4.8
V_90% _%	100%	96.6	99.8	95.0	97.2	97.8	72.3	76.8	98.6
V_107% _%	0%	16.7	0.0	8.2	4.5	0.9	41.6	37.3	5.3
SD Gy	0 Gy	0.3	0.1	0.3	0.3	0.2	1.0	0.7	0.2
CI	1.0	1.33	1.31	1.66	1.32	1.37	1.48	1.10	1.42

*PTV2 (50.4 Gy)*									
Mean Gy	50.4 Gy	50.7	51.0	50.8	50.7	50.4	50.4	50.4	50.6
Maxpt Gy	Prescript	56.8	52.2	54.1	56.9	52.9	53.2	52.1	55.5
D_1% _Gy	Prescript	55.4	52.0	53.3	-	52.4	52.4	51.8	54.2
D_99% _Gy	Prescript	44.8	48.0	45.7	-	47.2	47.3	48.7	45.7
V_90% _%	100%	98.5	100.0	99.2	-	100.0	99.9	100.0	99.5
V_107% _%	0%	8.5	0.0	0.0	-	0.0	0.0	0.0	1.5

*Vertebra*									
Mean Gy	< 20 Gy	15.1	17.8	16.5	19.0	16.8	19.2	20.8	19.0
									
**Spinal Cord**									
Max pt Gy	< 45 Gy	20.9	25.1	21.0	28.5	20.0	27.4	33.7	24.1
D_1% _Gy	Minimise	19.6	23.5	20.0	-	19.1	26.4	29.7	23.4
									
*Right Lung*									
Mean Gy	< 15 Gy	8.2	8.7	9.6	12.0	10.8	9.5	10.2	7.5
V_20 Gy _%	Minimise	15.0	17.3	20.6	-	24.1	20.9	21.5	12.4
									
*Left Lung*									
Mean Gy	< 15 Gy	10.9	12.9	13.2	14.4	14.5	11.1	12.4	11.1
V_20 Gy _%	Minimise	23.8	31.6	32.4	-	37.3	20.6	23.3	26.2
									
**Heart**									
Mean Gy	< 30 Gy	3.7	2.7	4.2	4.7	4.8	4.0	4.5	2.3
Max pt Gy	Minimise	50.2	49.0	49.8	41.4	44.9	37.3	42.6	30.5
D_1% _Gy	Minimise	45.5	40.5	48.3	-	42.6	35.4	41.7	24.8
									
**Healthy tissue**									
Mean Gy	Minimise	4.4	4.4	4.8	4.2	6.0	4.3	4.9	3.9
Max pt Gy	Minimise	55.1	49.9	53.5	53.3	50.3	50.7	50.0	54.5
V_10 Gy _cm^3^	Minimise	1020	1090	1250	-	1320	910	980	860
nV_10 Gy_	Minimise	8.8	10.0	10.0	-	12.9	7.9	8.5	8.4
V_90% _cm^3^	Minimise	20	16	35	-	29	13	28	26

^1^: TPS belonging to the first family type

^2^: TPS belonging to the second family type

**Table 7:** Results from dose plan analysis (total treatment) for Patient 3

	Obj	Corvus^1^	Eclipse^1^	Hyperion^2^	KonRad^1^	OMP^2^	PinnEUD^2^	PinnPhy^2^	Precise^2^
*PTV1 (45 Gy)*									
D_1% _Gy	Prescript	50.0	50.3	48.6	49.9	50.7	50.5	48.9	52.5
D_99% _Gy	Prescript	35.4	38.0	39.6	40.1	39.3	39.4	40.1	37.6
V_90% _%	100%	92.7	93.5	97.9	98.6	97.5	97.7	98.7	93.7
V_107% _%	0%	11.0	13.3	2.1	6.2	9.9	7.8	2.5	15.9
SD Gy	0 Gy	2.9	2.8	1.9	2.0	2.4	2.2	1.6	3.2
CI	1.0	1.78	1.04	1.49	1.53	1.67	1.80	1.85	1.48
									
*PTV2 (5.4 Gy)*									
D_1% _Gy	Prescript	5.9	5.6	5.8	6.1	6.0	9.7	7.5	5.8
D_99% _Gy	Prescript	4.6	4.9	5.0	4.6	4.7	1.0	1.6	4.7
V_90% _%	100%	96.8	99.1	99.5	97.7	97.9	53.4	74.1	98.3
V_107% _%	0%	4.2	0.0	1.2	13.6	4.3	44.4	47.3	2.1
SD Gy	0 Gy	0.2	0.1	0.2	0.3	0.2	2.1	1.3	0.2
CI	1.0	1.26	1.22	1.58	1.21	1.24	1.31	1.44	1.38

*PTV2 (50.4 Gy)*									
Mean Gy	50.4 Gy	50.0	50.3	50.3	50.2	50.1	50.0	50.3	50.3
Maxpt Gy	Prescript	57.6	57.5	54.2	56.3	56.7	58.0	56.8	61.6
D_1% _Gy	Prescript	55.4	55.7	53.0	-	55.7	56.0	55.2	58.6
D_99% _Gy	Prescript	40.8	42.5	45.0	-	44.1	42.5	43.0	43.6
V_90% _%	100%	92.8	93.8	98.7	-	97.0	92.8	96.6	96.2
V_107% _%	0%	6.6	9.1	0.0	-	9.0	9.4	4.0	11.4

									
**Uterus**									
Mean Gy	< 20 Gy	16.0	18.2	18.7	20.5	20.5	17.5	18.3	21.5
V_20 Gy _%	Minimise	4.2	16.4	38.9	-	49.6	7.5	13.4	53.5
									
**Rectum**									
Mean Gy	< 40 Gy	19.0	21.2	27.4	27.3	25.4	21.7	24.7	21.8
V_40 Gy _%	Minimise	2.3	12.1	26.7	-	21.9	13.5	14.2	16.8
									
**Bladder**									
Mean Gy	< 30 Gy	24.2	25.6	24.0	27.3	30.0	18.0	21.2	25.1
V_30 Gy _%	Minimise	4.5	22.5	12.8	-	49.1	6.0	5.1	18.5
									
**Right Femur**									
Mean Gy	< 20 Gy	18.3	20.6	19.7	24.9	19.6	15.0	15.2	20.2
Max pt Gy	Minimise	40.4	43.6	39.4	43.7	46.5	37.6	38.8	35.9
									
*Left Femur*									
Mean Gy	< 20 Gy	17.0	19.4	19.0	23.6	18.9	15.7	15.8	20.0
Max pt Gy	Minimise	39.5	39.8	40.3	42.2	40.7	30.2	34.4	40.0
									
**Healthy tissue**									
Mean Gy	Minimise	13.9	11.3	13.3	16.9	13.8	14.1	14.3	11.8
Max pt Gy	Minimise	59.1	52.6	50.7	57.5	56.8	58.0	58.6	62.6
V_10 Gy _cm^3^	Minimise	3630	3190	3750	-	3750	3790	3840	3400
nV_10 Gy_	Minimise	5.8	5.2	5.9	-	6.3	5.9	6.0	5.5
V_90% _cm^3^	Minimise	328	22	123	-	78	319	264	190

^1^: TPS belonging to the first family type

^2^: TPS belonging to the second family type

**Table 8:** Results from dose plan analysis for Patient 4

	Obj	Corvus^1^	Eclipse^1^	Hyperion^2^	KonRad^1^	OMP^2^	PinnEUD^2^	PinnPhy^2^	Precise^2^
*PTV1 (18 Gy)*									
Maxpt Gy	Prescript	21.0	20.0	21.4	22.5	21.7	23.9	21.0	23.6
D_1% _Gy	Prescript	20.0	19.5	20.4	21.6	19.8	21.7	19.6	21.4
D_99% _Gy	Prescript	15.2	14.2	11.8	12.9	16.2	14.5	16.2	14.3
V_90% _%	100%	96.8	96.7	91.9	95.2	99.0	97.4	99.0	97.6
V_107% _%	0%	7.3	2.4	14.6	18.5	3.8	13.8	2.2	12.7
SD Gy	0 Gy	0.9	0.9	1.5	1.5	0.7	1.2	0.6	1.2
CI	1.0	1.53	1.12	1.11	1.30	1.28	1.16	1.27	1.31

*Kidney*									
Mean Gy	< 10 Gy	7.7	5.1	6.4	8.0	9.3	7.7	9.7	9.3
Max pt Gy	Minimise	17.5	13.4	12.2	13.4	18.1	13.7	17.3	14.1
D_1% _Gy	Minimise	14.8	12.0	11.3	12.5	16.7	12.7	16.0	13.4
V_5 Gy _%	Minimise	78.6	39.7	72.2	97.2	98.0	87.8	97.3	98.1
									
**Healthy tissue**									
Mean Gy	Minimise	8.8	6.1	7.0	7.9	8.2	7.1	7.6	7.0
Max pt Gy	Minimise	20.5	19.7	17.7	22.6	21.6	20.6	20.6	23.1
V_10 Gy _cm^3^	Minimise	1920	970	1200	1580	1650	1380	1600	1300
nV_10 Gy_	Minimise	1.9	0.8	0.9	1.3	1.4	1.1	1.3	1.1
V_90% _cm^3^	Minimise	561	206	219	440	339	227	331	348

^1^: TPS belonging to the first family type

^2^: TPS belonging to the second family type

In all Figures, the KonRad data are shown only for the last patient while in the tables, the results are shown only for the mean and maximum point doses for the summed plans since dose distributions could not be summed up, as described above.

### Target coverage

For PTV1 and PTV2 the analysis was conducted also for the DVHs of the separate courses. In this case, the results for the TPS of the second family, are poorer for the boost for the reason described in the methods (CI, in some cases, e.g. Patient 1, is even lower than 1). This feature also affects the results in table 4 which shall therefore be considered with some caution for Hyperion, OMP, Pinnacle^3 ^and Precise (e.g. CI).

Analysing the data, it is possible to notice certain uniformity of results for most of the parameters. In some cases, these are all sub-optimally fitting the objectives and prove the difficulty of all the TPS to achieve high conformality on targets when, as for paediatric cases, the fulfilment of dose constraints for OARs and HTis is emphasised. The risk of under dosage of the PTV is common to all TPS (e.g., from table 4 and complementary tables, V_90% _and D_99% _present large deviations from the ideal objective values). For Patient 1, PinnEUD showed a large over dosage of the PTV2 (total) where V_107% _= 23% (table 5); this is significantly different from all other cases. For the two most complicated cases, OMP showed the best values for patient 1 (difficult for the small superficial scar volume), and Hyperion for patient 3 (difficult for the positioning of the three PTVs with particularly radiosensitive OARs in between).

### Organs at risk

Given the different anatomical location of the tumours and the different PObj for each OAR, each of the 4 patients is considered separately.

Patient 1: the objective selected for the vertebra (that was partially included in the target) was respected only by OMP (table 5) (and almost by Hyperion). Doses larger than 25 Gy were observed for Precise and PinnPhy. The PObj for spinal cord was only not reached by PinnPhy (looking at the maximum point dose) but the limit was not violated if D_1% _is considered. All TPS respected the constraint on the mean dose to contra lateral lung and Hyperion was the only TPS to (almost) keep the mean dose to the uninvolved omolateral lung below 15 Gy. KonRad was the only TPS not able to reach the objective for the heart. Averaging over the TPS, the PObj were not respected for the vertebra and for the uninvolved omolateral lung (table 3).

Patient 2: PObj's were respected by all TPS, with the minor exception of PinnPhy where the mean dose to the vertebra was 20.8 Gy instead of 20 Gy.

Patient 3: From table 3, on average, all objectives were respected. For the mean uterus dose of 20 Gy, Precise (21.5 Gy), KonRad (20.5 Gy) and OMP (20.5 Gy) show minor violations. Bladder and Rectum did not cause any problems (OMP reached the limit on the bladder; Hyperion and OMP presented V_40 Gy _larger than 20% in the rectum). The mean dose to the femurs was violated quite substantially by KonRad (both left and right femurs); Eclipse showed a small deviation for the right femur; Precise was at the limit for both femurs.

Patient 4: the only OAR considered in this plan was the right kidney, the only one in the patient (nephrectomy had been performed to the left kidney). The PObj was fixed very conservatively and a very high priority was assigned to this organ during optimisation. All systems respected the objective and the best values (in terms of mean dose and V_5 Gy_) were reached by Eclipse (followed by Hyperion).

### Healthy tissue sparing

In the tables are reported, for HTis, the mean dose, the maximum point dose, V_10 Gy_, nV_10 Gy _and V_90% _(to analyse the presence of hot spots). KonRad was not completely analysed (only mean and max doses are reported) except for patient 4. Regarding V_10 Gy_, the best results were achieved by Hyperion and Eclipse (patient 1), Precise and Pinnacle^3 ^(patient 2), Eclipse (patients 3 and 4). V_10 Gy _is interpreted as a global dose bath, and the mean values ranged from 1060 ± 170 cm^3 ^(patient 2) to 3620 ± 240 cm^3 ^(patient 3). The value of nV_10 Gy _ranged from 1.2 (patient 4) to 9.5 (patient 2) if averaged over the TPS, and from 5.0 for PinnEUD and Eclipse to 6.4 for OMP if averaged over the patients.

The average values of V_90% _ranged from 24 ± 8 cm^3 ^(patient 2) to 334 ± 122 cm^3 ^(patient 4). The best results were for Corvus (patient 1), PinnEUD (patient 2), Eclipse (patients 3 and 4).

Considering the qualitative evaluation of dose distributions of figures 1 and 2 and the DVH of the figures 3, 4, 5, 6, it is clear, e.g. patients 3 and 4, that the high sparing of OARs reached by Pinnacle^3 ^was counterbalanced by a poorer management of the HTis. For the fourth patient, the best sparing of the kidney obtained by Eclipse and Hyperion was associated with a consistently better management of the HTis and a relatively low dose bath. The effect (e.g. for Eclipse) is quite visible in the DVH of figure 6.

### MU evaluation

MU/Gy are summarised in table 9. Over all TPS a mean value of 256 MU/Gy has been determined, about twice that of a 3DCRT plan. Precise and KonRad present the lowest values, while Corvus and Eclipse the highest. The use of the dynamic sliding window delivery (Eclipse) is not significantly worse than the static segmental (step and shoot) technique in this regard.

**Table 9:** MU/Gy for all TPS averaged over all patients (mean ± SD, [range]).

TPS	MU/Gy
Corvus	344 ± 174 [167, 648]
Eclipse	337 ± 188 [162, 684]
Hyperion	263 ± 129 [136, 513]
KonRad	191 ± 64 [145, 294]
OMP	263 ± 121 [149, 503]
PinnEUD	220 ± 86 [133, 316]
PinnPhy	238 ± 54 [172, 307]
Precise	190 ± 73 [133, 343]

### 'Global plan quality'

The findings for the Score parameters are reported in table 10 for all TPS individually for the single patients and averaged over the patients (uncertainty is given at one SD). The lowest (better) average value was achieved for OARs by Eclipse (0.75), the highest (worst) by PinnPhy (0.92), for targets the best results were achieved by PinnPhy and OMP (0.05 and 0.06).

**Table 10:** Global scores for the treatment plans. Scores for OARs and PTVs should be as low as possible and not larger than 1.

		Corvus^1^	Eclipse^1^	Hyperion^2^	KonRad^1^	OMP^2^	PinnEUD^2^	PinnPhy^2^	Precise^2^
Patient 1	OARs	0.97	0.89	0.82	0.99	0.89	0.93	1.10	1.05
	Targets	0.26	0.15	0.13	0.15	0.08	0.25	0.12	0.25
									
Patient 2	OARs	0.60	0.67	0.69	0.70	0.73	0.66	0.73	0.60
	Targets	0.13	0.00	0.03	0.08	0.00	0.00	0.00	0.03
									
Patient 3	OARs	0.81	0.86	0.90	1.01	0.94	0.77	0.81	0.90
	Targets	0.16	0.18	0.03	0.12	0.12	0.13	0.06	0.19
									
Patient 4	OARs	1.05	0.59	0.73	0.80	1.03	0.86	1.04	0.91
	Targets	0.11	0.06	0.23	0.23	0.05	0.16	0.03	0.15

Mean ± SD	OARs	0.86 ± 0.20	0.75 ± 0.15	0.79 ± 0.09	0.88 ± 0.15	0.90 ± 0.13	0.80 ± 0.12	0.92 ± 0.18	0.87 ± 0.19
	Targets	0.16 ± 0.07	0.10 ± 0.08	0.10 ± 0.10	0.14 ± 0.07	0.06 ± 0.05	0.14 ± 0.05	0.05 ± 0.05	0.15 ± 0.09

^1^: TPS belonging to the first family type

^2^: TPS belonging to the second family type

## Discussion

The study aimed to address the effectiveness of IMRT treatment planning on various paediatric indications. The study compared eight TPS with a common data set and planning guidelines, reproducing the model already adopted in a previous study on breast treatment [20]. The complexity of IMRT planning on paediatric patients was confirmed by the study. Differences in plans from various TPS, both in terms of PTV coverage and OAR sparing, were observed. Care has to be taken in ranking the TPS, since the influence of user preferences on the planning results has to be considered too: where goals cannot be achieved simultaneously, some trade-off has to be found that satisfies the individual planner. In this context, the mean scores do allow an assessment of both the TPS quality and the user preferences.

Each patient case was selected as paradigmatic of some planning challenge (exemplified in the methods) in combination with very strict dose constraints deriving form the paediatric environment. All plans sufficiently respected objectives and won challenges, therefore the general conclusion is that modern optimisation algorithms can technically succeed in managing very restrictive conditions and are in principle considerable for application in paediatric practice. Detailed studies on individual paediatric pathologies could provide more quantitative information on specific questions (with all the complications arising from inter-patient variability present in paediatrics) and statistically substantiate our present proof of principles but this is a target that can be achieved also in more common pathologies (as in the case of breast for adults [20]) whereas the fundamental question of understanding basic response of the main commercial systems to paediatric IMRT is addressed by this study.

Considering target coverage and limiting the discussion to the second family of TPS, significantly heterogeneous dose distributions were observed for the targets in the boost courses. Considering as an example (without any implication of merit) PinnEUD and patient 1, for PTV2, the volume receiving less than 90% of the prescribed dose (and dose/fraction) was about 31% (i.e.~1/3 of the PTV received a dose per fraction lower than prescription), similarly V_107% _was about 54% (~1/2 of the PTV received a dose per fraction higher than prescription); as a consequence only ~1/6 of the PTV would receive the prescribed dose per fraction (within 90% and 107%). The biological and clinical response of one third of the target volume receiving low dose per fraction could raise some issue about local control probability. This effect derived directly from the optimisation and planning strategies implemented for boost volumes that included the knowledge of dose distributions computed for the previous course, that in principle should try to compensate hot and cold spots. This effect is enhanced by the fact that the boost dose is significantly smaller than the dose prescribed to PTV1. Hence, attention should be paid to keep the dose per fraction within certain limits if hot and/or cold spots should be compensated (for example, in Hyperion, plans were optimised with a mixture of dose per fraction and total dose objectives).

The previous example clarifies extremely well the absolute importance for centres willing to approach IMRT (not only in paediatrics) to establish precise treatment protocols, including fractionation issues and, depending on the TPS available, it could be necessary to adapt historical traditions to technological constraints (this is very important especially when simultaneous integrated boost techniques are under discussion).

In the paediatric treatments, great care has to be taken on OARs, and in the present study, the effort was put to have better sparing; in some cases, this could lead to more dose heterogeneity in the target. This is an issue that has to be pursued with adequate objective definitions in the paediatric field using dose constraints specific for paediatric patients, from proper publications, which are significantly different (and more demanding) from what normally used for adults. Therefore, the results presented are appropriate to appraise the performances of different TPS under the severe restrictions that shall be applied for paediatric IMRT and not as a simple measure of TPS reliability for IMRT, proven elsewhere [20]. It is obvious that, the more restrictive the planning objectives are, the more difficult the optimisation process should be. Nevertheless, the qualitative pattern of results from the different TPS is encouraging towards a possible usage in paediatric clinical practice.

Another important point is linked to the analysis of the HTis involvement. Assuming the need to reduce maximally the amount of HTis irradiated at any dose level, no system was able to achieve the goal. Considering nV_10 Gy_, it resulted in average at least 5 times greater than the target volume (excluding patient 4 where the prescribed dose was anyway low). In addition, a large variation was observed among systems. Looking at the standard deviation reported for V_10 Gy _in table 3, this ranged from 7% (240 cm^3^) for patient 3 to 21% (300 cm^3^) for patient 4. This variability among TPS should be seriously considered when IMRT treatments are prescribed to children. The TPS industry should be urged to introduce efficient tools to cope with this need in their products, and some improvements appear to be coming from research developments and pre-clinical releases. Nevertheless, it is fundamentally impossible with photons to avoid the presence of rather extended dose baths.

In terms of organs at risk, the study is affected by some limitations that deserve clarification. For patient 2, breasts were not explicitly included in the list of organs at risk and for patient 3 the same applies to ovaries. Concerning breast, in this case the issue is not heavy involvement of the glands (i.e. irradiation at high dose levels) but rather the dose bath and the potential for secondary cancer induction. In the absence of reliable models to predict the risk of secondary cancers (unfortunately all the studies that appeared so far are not conclusive) it would be only speculative to show data pointing to this endpoint. Moreover there are no values in literature that can reliably be used as tolerance dose levels for breast irradiation (as an organ) in children. Nevertheless, breasts were anyway included in our analysis as part of the healthy tissue (instead of considering them as specific organs) and therefore they were accounted for in the results presented for generic healthy tissue.

Concerning ovaries, this is an even more delicate case since dose tolerance (in the range of 4–12 Gy) changes, decreasing, with increasing age. In principle it would be appropriate to include these organs in the analysis but, in the specific case, it was difficult to properly identify ovaries on the available CT dataset. The only viable solution was, at the limit, to draw a likelihood region probably including the ovaries. From the analysis point of view, given the tolerance level very low with respect to the prescribed dose (50.4 Gy), given the impossibility to have a correct location of the organs, and given their close proximity to the target, it would be hard to quote reliable predicted dose values for these organs to appraise IMRT performances on an improperly defined object. Furthermore, the objective to keep the ovarian dose below 10% of the prescribed dose (that is the tolerance level) is essentially impossible with standard IMRT approaches for physical reasons (scattering, penumbras and dose gradients). Since the ovaries are basically 'surrounded' by the target; the trade-off between sufficient sparing and target under dosage would be hardly acceptable. This means that, in addition to the delineation problems mentioned above, and in the present study at least, it was considered as unavoidable to renounce to ovaries as a primary organs at risk and we were forced to accept their compromission. To give anyway a qualitative appraisal of the different systems, we recorded the dose to the 'ovarian region' resulting from the plans in the study, and this resulted to be of the order of 30 Gy regardless from the TPS in the slices where it was possible to roughly identify them. The lesson to learn from this case is that, for IMRT in general and particularly for pediatrics, high spatial resolution and the eventual need for combined imaging modalities or contrast agents should be considered as a priority for target delineation and therefore we should all systematically change our normal codes of practice (with also potential financial implication depending on the reimbursement schemes).

As proven in many other IMRT studies, the degree of conformal avoidance with photon based IMRT is not perfect and trade-offs should be considered for complex situations. This proves to be particularly important in the case of paediatric oncology due to low tolerances, small distances among organs, growth problems and risk of secondary cancer induction are all concurring elements that could increase the interest for alternative treatment modalities. In particular, the usage of protons, which is completely out of the aim of our study, should be mentioned as particularly interesting due to the excellent physical properties (lateral limited scattering and sharp fall-off of doses at distal edge of the Bragg's peaks). Some studies appeared and potentials are encouraging [23] even if care should be put on the proton technique as pointed out by Hall [19] since, for example, passive scattering modalities could increase the neutron contaminations and the "MU" needed to delivery the prescribed dose with potential impact on the probability of secondary cancer induction. More general and widespread comparative studies should be necessary to identify proper indications but, in the absence of generally available proton facilities, or in the presence of severe logistic limits, photons based IMRT could be anyway considered as a valid approach.

The stability or sensitivity of different TPS against variations in the planning objectives was not considered as part of the study because considered as beyond the purposes of the study and would deserve a dedicated study, regardless from paediatric indications. Furthermore, it would be extremely complicate to perform correctly a similar study since we used, to define the study strategies, "clinical" planning objective rather the specific dose-constraints which are depending from the individual TPS and their optimisation methods. The translation of fluctuations in clinical objectives into fluctuations of TPS-depending constraints would be affected by intrinsic uncertainties probably masking the sensitivity effects to investigate.

The accuracy of the dose calculations (against measurements) was not considered in the study, because it is seen as part of a proper commissioning of IMRT and was already partially discussed in [20]. Notice that the different dose calculation algorithms used can be divided into two groups: the PB related algorithms, and the 'advanced' ones like CC, AAA and MC, where the lateral electron transport is taken into account, giving more reliable results in heterogeneous media (especially in low density tissues) and possibly in HTis. In this way the study incorporated in an indirect way the impact of dose calculation algorithms on IMRT planning comparisons. In general, we consider appropriate to evaluate globally the performances of different TPS without explicitly correcting for (known) limitations in dose calculation engines since in this way it is possible to reproduce more precisely potential clinical conditions. In addition to this, it would be substantially impossible to disentangle the optimisation phase from the dose calculation engines. An ideal procedure would consist of comparing TPS limiting to the optimisation phase and performing the final dose calculations using only one (e.g. MC) reliable engine. In fact, for most of the TPS this is impossible because, if the optimisation is performed using pencil beam algorithms (eventually simplified for speed reasons), the multileaf segmentation engines quite often include some consideration of the head scattered radiation from the linac head and this is intimately connected with the final dose calculation engines. Therefore no true factorisation process is possible to limit a comparison of performances to the optimisation phase. On the other side, the impact of dose calculation algorithms in some range of clinical conditions is object of independent evaluations in more standard conditions (e.g. Knöös [24]) and results can be likely generalised to IMRT.

The issue of MU was here addressed simply analysing the MU/Gy for all TPSs, even if no effort was put in the application of models to estimate secondary cancer induction from the observed 3D dose distributions. This is an important aspect of paediatric radiation oncology, and detailed descriptions of linac head, shielding, beam spectra, neutron and electron contamination should be modelled in the dose calculation algorithms. This was felt to be beyond this study; meanwhile it should be considered a mandatory code of good practice to maximise the efforts to keep the delivered MU to young patients as low as possible to minimise the risk of inducing secondary malignancies. The average value of MU/Gy observed in the present study is not significantly different from what reported in the breast study for the same systems in both relative and absolute terms proving a good stability of systems and their performance independence from the challenge to solve. In this respect, the plans analysed in the present paper show that IMRT may be efficiently used in paediatric patients increasing to a certain extent the risk of second cancer (about doubled with respect to 3DCRT [19]). It is however well known that the number of fields, the modulation degree, the number of IL and segments may influence the MU/Gy delivered with IMRT plans.

## Conclusion

Eight TPS were compared to assess the capability to plan IMRT in different paediatric patients. All the TPS allowed the design of plans mostly respecting initial objectives even if with a range of differences. Emphasis should be made of the importance of avoiding hot spots outside targets and in the maximal reduction of HTis involvement. This normal tissue and OAR sparing leads inevitably to more heterogeneity in the target dose distribution. Some systems provided better capabilities (as measured by the scoring indexes), within the limits of user preferences, than others but performance should be evaluated case by case according to clinical requirements and strategies. The key message concerning the possibility to consider IMRT for paediatric treatments is that all systems proved to offer sufficient performances from the technical point of view. Concerns remain about the relevance of large dose baths, not avoidable with IMRT in this class of patients.

## Competing interests

No special competing interest exists for any authors.

Dr. Mats Asell is employed by Nucletron AB and is in the development group of Oncentra Masterplan one of the systems used in the study.

Dr. Malin Larsson is employed by RaySearch Laboratories (Stockholm, Uppsala) and is in the development group of the optimisation algorithms implemented in both Philips Pinnacle and Nucletron Oncentra Masterplan used in the study.

## Authors' contributions

AF and LC designed the study.

AF, GN, FL, MA and BD defined planning protocols and operative procedures.

RW defined volumes of interest.

MA performed planning on Masterplan.

ML performed planning on Pinnacle.

AF and GN performed planning on Eclipse.

JM and MA performed planning on Hyperion.

BD, FL and FL performed planning on Konrad.

MP and FL performed planning on PrecisePlan.

BD, DW and FL performed planning on Corvus.

AC, EV, AF and GN coordinated and carried out data collection, program development and statistical analysis

LC wrote the manuscript.

All authors contributed read and approved the final manuscript.
